# Phosphate Modified Screen Printed Electrodes by LIFT Treatment for Glucose Detection

**DOI:** 10.3390/bios8040091

**Published:** 2018-10-16

**Authors:** Francesco Milano, Livia Giotta, Daniela Chirizzi, Simos Papazoglou, Christina Kryou, Annarita De Bartolomeo, Vincenzo De Leo, Maria Rachele Guascito, Ioanna Zergioti

**Affiliations:** 1 Istituto per i Processi Chimico Fisici, UOS Bari, Via Orabona 4, 70126 Bari, Italy; francesco.milano@cnr.it (F.M.); v.deleo@ba.ipcf.cnr.it (V.D.L.); 2 Dipartimento di Scienze e Tecnologie Biologiche e Ambientali, Università del Salento, S.P. Lecce-Monteroni, 73100 Lecce, Italy; livia.giotta@unisalento.it; 3 IZS Puglia e Basilicata, U.O. Putignano. Via Chiancolla 1, C.da. S. Pietro Piturno, 70017 Putignano (BA), Italy; daniela.chirizzi@izspb.it; 4 Department of Physics, National Technical University of Athens, Iroon Polytehneiou 9, Zografou, 15780 Athens, Greece; simpap@mail.ntua.gr (S.P.); chkryou@central.ntua.gr (C.K.); zergioti@central.ntua.gr (I.Z.); 5 Dipartimento di Beni Culturali, Università del Salento, S.P. Lecce-Monteroni, 73100 Lecce, Italy; annarita.debartolomeo@unisalento.it; 6 Dipartimento di Chimica, Università di Bari “Aldo Moro”, Via Orabona 4, 70125 Bari, Italy

**Keywords:** screen printed electrodes, laser printing, LIFT, glucose, biosensor

## Abstract

The design of new materials as active layers is important for electrochemical sensor and biosensor development. Among the techniques for the modification and functionalization of electrodes, the laser induced forward transfer (LIFT) has emerged as a powerful physisorption method for the deposition of various materials (even labile materials like enzymes) that results in intimate and stable contact with target surface. In this work, Pt, Au, and glassy carbon screen printed electrodes (SPEs) treated by LIFT with phosphate buffer have been characterized by scanning electron microscopy and atomic force microscopy to reveal a flattening effect of all surfaces. The electrochemical characterization by cyclic voltammetry shows significant differences depending on the electrode material. The electroactivity of Au is reduced while that of glassy carbon and Pt is greatly enhanced. In particular, the electrochemical behavior of a phosphate LIFT treated Pt showed a marked enrichment of hydrogen adsorbed layer, suggesting an elevated electrocatalytic activity towards glucose oxidation. When Pt electrodes modified in this way were used as an effective glucose sensor, a 1–10 mM linear response and a 10 µM detection limit were obtained. A possible role of phosphate that was securely immobilized on a Pt surface, as evidenced by XPS analysis, enhancing the glucose electrooxidation is discussed.

## 1. Introduction

The electro-oxidation of glucose has been studied for a number of years with the aim of developing reliable sensors for the detection of glucose levels in blood, both in vitro and in vivo, and/or implantable safe bio-fuel cells [1]. Glucose detection, based on electrochemical (bio)-sensing technology, has attracted more and more interest owing to its elevated selectivity, accuracy, repeatability, and low cost [2]. Related literature is extensive, both for enzymatic [3] and non-enzymatic [4] sensors. Although glucose electro-oxidation has been studied at a variety of electrode materials as well as modified electrodes [3], most investigations have employed either gold or platinum [5] as electroactive species with a particular focus on their use as micro- and nano-structured composite materials [6], often as an alternative to the most labile enzyme based bio-sensors counterpart [5].

Screen printing 2D technology is widely used to mass produce disposable sensor chips based on screen printed electrodes (SPEs) as a valid technological alternative to traditional 3D electrodes. This technology is privileged for its inexpensive costs, well-established process development, fast production line, and high manufacturing capacity. Their compact 2D shape makes them suitable for batch, flow injection analysis, and as electrochemical detectors for high-performance liquid chromatography. Specific pretreatments for improving SPE electrochemical properties have been reported principally for C based SPEs that use a very cheap bulk material. For example, effects of the SPE treating with *N,N*-dimethylformamide (DMF) solvent have been recently reported and extensively discussed [7], using a range of electroactive compounds (i.e., hexaammineruthenium(III) chloride, the reduced form of dihydronicotinamide adenine dinucleotide and capsaicin). For the electroanalytical sensing of dihydronicotinamide adenine dinucleotide and capsaicin, increments of only 1.08-fold and 1.38-fold were respectively observed relative to unmodified SPEs. These results were not in line with previous results, showing that DMF was much more effective in enhancing the electrochemical performance of screen printed electrodes, attributing this effect to the dissolution of the SPE working electrode binder [8]. Based on literature data, care must be taken to prevent some effects of SPEs degradation observed when using DMF [7]. Besides, few efforts have been reported for Pt and Au based SPEs to improve their electrochemical performances in specific applications (i.e., potential use in room temperature ionic liquids [9]), by comparing different pretreatment methods (i.e., SPE pretreated by NaOH, DMF, tetrahydrofuran, and mechanical polishing) [10]. However, the chemical and electrochemical activation procedures of electrode surfaces must be repeated before each use. In the case of SPEs, that are supposed to be ready for use, the pre-activation should be avoided by the final user, and should be ideally carried out by the manufacturer using a procedure with permanent effect.

**Scheme 1:**
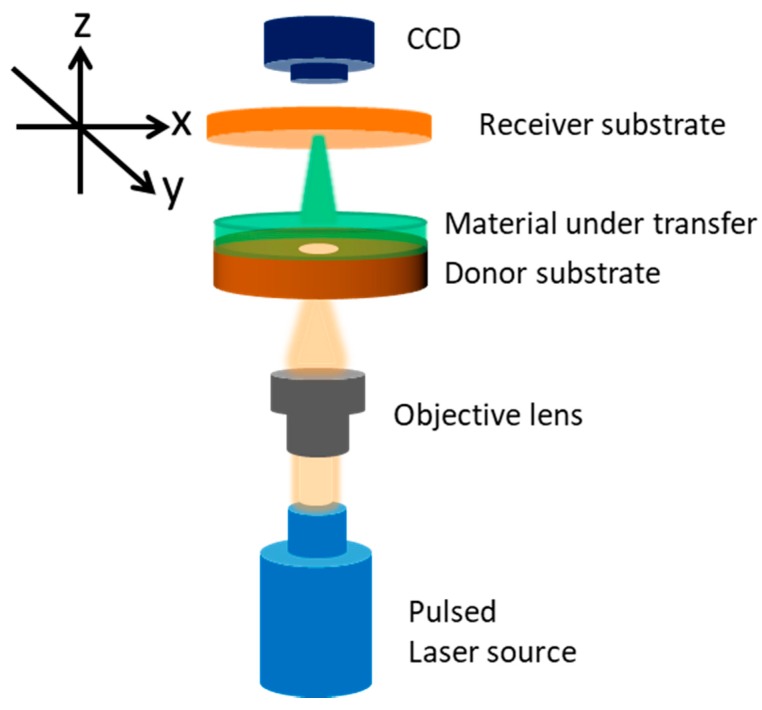
Schematic illustration of the laser induced forward transfer (LIFT) experimental setup.

Laser induced forward transfer (LIFT) is a direct printing technique that was first introduced in 1986 by Bohandy et al. [11] for the transfer of copper on silicon substrates. Since then, it has rapidly expanded in the fields of electronics and biosensors for the transfer of a wide variety of organic and inorganic materials. As depicted in Scheme 1, the technique involves two substrates, namely the donor and the receiver substrates, which are brought in close proximity and parallel to each other. The donor substrate is usually transparent (quartz) and is coated with the material under transfer. Often, a thin metallic layer (Ti) is deposited first on one side of the donor substrate and acts as an absorbing or sacrificial layer so as to absorb the incident laser pulse and trigger the ejection mechanism. As the laser pulse irradiates the donor substrate from the back side (quartz) and is being focused at the interface between the sacrificial layer and the material under transfer, the temperature rapidly and locally rises and this leads to the creation of a directional jet of the material that travels towards the receiver substrate. In this way, digitally controlled patterns (droplet matrices, lines, complex features) of materials may be formed, using a charge-coupled device camera and a motorized micromanipulator to move the receiver substrate. The main advantages of the technique are the high spatial resolution that lies in the micrometer regime and the rapid and precise deposition of material patterns on substrates that include rigid (silicon, SiO_2_) and flexible polymeric surfaces.

In this paper, we investigated the use of LIFT technique as a pretreatment of Pt, Au, and glassy carbon (GC) SPEs to immobilize phosphate saline droplets on a working electrode so that the electrochemical performances of the SPEs are modified. The co-immobilization of phosphate is a common occurrence when LIFT is employed for obtaining electrodes functionalized with biological materials that often require neutral pH buffering. It is thus interesting to assess the role of phosphate in inducing electrode surface modifications by itself. The treated electrodes have been characterized morphologically by atomic force microscopy (AFM) and scanning electron microscopy (SEM) and chemically by X-ray photoelectron spectroscopy (XPS). Interestingly LIFT phosphate treatment heavily influences the electrochemical properties of all tested surfaces. In the case of gold, we found a radically reduced electrochemical activity, while in the case of GC and Pt was drastically increased. These results led us to try the activated Pt surface as a non-enzymatic glucose sensor, in which the glucose is oxidized directly on the electrode surface. The enhanced electrochemical activity of Pt SPE modified surfaces makes them attractive as a platform for further functionalization with other materials as biomolecules in (bio)-sensing [12,13,14,15] and/or fuel cell applications.

## 2. Materials and Methods

All chemicals of the highest purity were used without further purification. Ferrocyanide ([Fe(CN_6_)]^4−^), ferricyanide ([Fe(CN)_6_]^3−^), reagent grade phosphate buffers and glucose were purchased from Sigma. All aqueous solutions were prepared using water obtained by Milli-Q Gradient A-10 system (Millipore, 18.2 MΩ cm, organic carbon content ≤4 μg/L).

Scanning electron microscopy, AFM and XPS were used to characterize the morphology and the chemical structure of commercial screen printed Pt working electrodes before and after modification by LIFT. Scanning electron microscopy and AFM were used to characterize the morphology of commercial Au and GC SPEs as purchased and after LIFT treatment.

Scanning electron microscopy was carried out by means of a field emission FEI NanoSEM 230 A system, attached to an Everhart-Thornley detector. Scanning electron micrographs were recorded in a high-vacuum mode with electron accelerating voltage of 2 kV.

The morphology of the obtained LIFT treated electrode surfaces was studied using an AFM (Veeco Innova, Athens, Greece) operated in contact mode.

X-ray photoelectron spectroscopy analysis was carried out using an AXIS ULTRA DLD (Kratos Analytical spectrometer, Kyoto, Japan), with an Al Kα monochromatic radiation (1486.6 eV) source, operating at 15 kV and 15 mA. The pressure in the analysis chamber was 3 × 10^−9^ torr. Survey spectra and high resolution (HR) regions were acquired in fixed analyzer transmission (FAT) mode, with survey spectra at pass energy E_0_ of 160 eV and energy step of 1 eV, and HR spectra at E_0_ of 20 eV and energy step of 0.1 eV. A hybrid lens mode was used for all measurements with analysis area of about 700 μm × 300 μm. During data acquisition, a system of neutralization of the charge has been used. Curve-fitting of photoelectronic peaks was performed by means of software New Googly [16] which allows satellites and background correction. Peaks were assigned by comparing the binding energy (uncertainty of ± 0.1 eV) to that found in literature and also in the standard reference database of the National Institute of Standard and Technology [17]. In our work, the C 1s aliphatic carbon peak at 285.0 eV [18] was used as an internal standard.

The LIFT modification of the screen printed working electrode (WE) was carried out by means of an experimental setup described elsewhere [15,19]. In the experiment, 10 μL of water or phosphate buffer 0.1 and 1 M pH 7.0 was drop-cast on a donor substrate that was selectively deposited on the receiver substrate (the WE of the SPE), positioned parallel, in close proximity (300 μm) to the donor substrate. Two-dimensional patterns of buffer droplets, each deposited by a single laser pulse, were printed on the receiver substrate. In this work, a pulsed nanosecond laser operating at 355 nm (1 Hz) with a fluence of 470 mJ/cm^2^ was employed and focused using a laser objective lens (15×, 13 mm working distance) to generate a laser spot with diameter of 80 μm, as measured on the thin Ti layer which was used to absorb the laser light. The droplets diameter on the receiver substrate was 150 μm. The WE surface was uniformly modified by adjusting the separation distance of the droplets.

Electrochemical experiments were carried out by means of a µStat400 portable electrochemical sensor interface workstation (DropSens, Spain) controlled by a computer. DRP-220AT (gold), DRP-550 (platinum), DRP-110 (GC) SPEs and relevant connectors were also from DropSens. The SPEs ceramic support was L33 × W10 × H0.5 mm; the WE (4 mm diameter) was partially surrounded by a silver quasi-reference electrode, while the counter electrode (made up of gold, platinum or GC) was the outermost ring of the device. The medium used for electrochemical measurements was 90 mM phosphate buffer (pH 7.0) or 0.4 M KCl and the electrode was immersed in a cylindrical vessel filled with 9 mL of electrolyte solution saturated in N_2_. Glucose was added from a stock solution 1 M, spanning concentrations from 1 to 100 mM.

## 3. Results and Discussion

### 3.1. LIFT Treatment of Pt and Response to Glucose

#### 3.1.1. Morphological and Chemical Characterization

**Figure 1:**
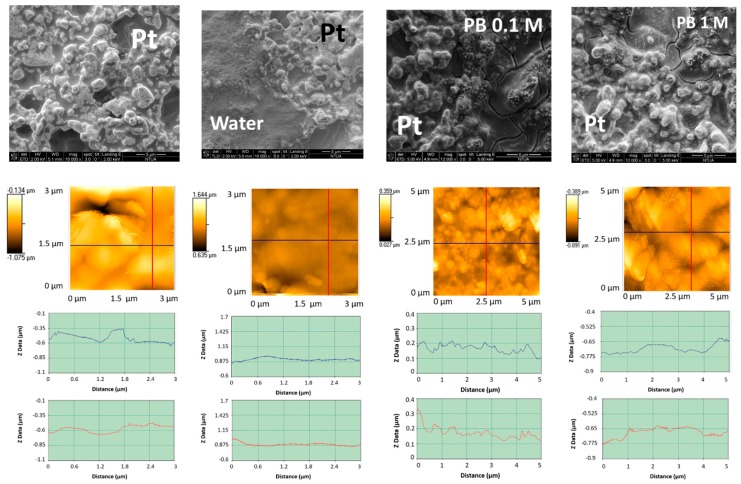
First row: SEM (from left to right) of untreated Pt and LIFT treated with water, phosphate buffer 0.1 M and phosphate buffer 1 M; second row: the corresponding topographic atomic force micrographs; third row: roughness profiles in the two orthogonal directions indicated in the atomic force micrographs.

The first row of Figure 1 shows the scanning electron micrographs (from left to right) of an untreated Pt working electrode and those treated by LIFT with water, 0.1 M phosphate buffer, and 1 M phosphate buffer. The corresponding atomic force micrographs are presented in the second row of the same figure. The mean roughness was found to decrease from 130 nm to 52 nm after water treatment and the results were substantially similar with 0.1 or 1 M phosphate buffer. The LIFT treatment induced a flattening of the Pt surface, possibly due to the impact of the water droplets. Moreover, the sample irradiation with the laser in the same conditions but with a bare donor substrate resulted in a roughness decrease to 90 nm (not shown) that in this case can be attributed to the thermal effect of the laser pulse on the receiver surface.

All Pt SPEs samples were analyzed using XPS to characterize the chemical composition of the electrode surfaces. X-ray photoelectron spectroscopy was chosen since it probes the composition of the external few nanometers of materials and the electrochemical activity strictly depends on the outermost atomic layers in contact with the electrolytic solution.

**Figure 2:**
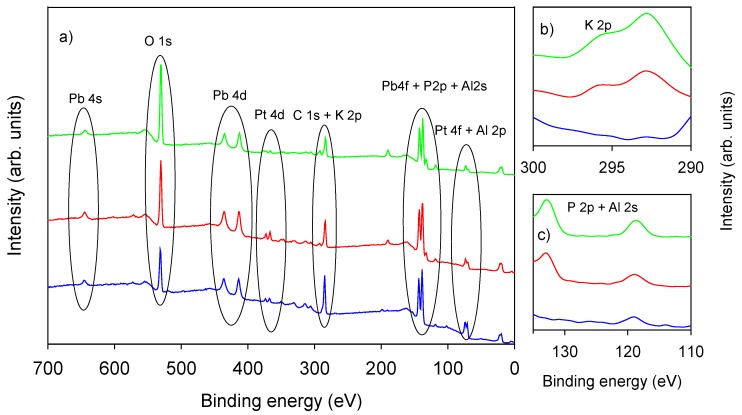
(**a**) XPS survey of untreated Pt SPE (blue trace) and of LIFT treated screen printed electrodes (SPEs) with phosphate buffer 0.1 M (red trace) and 1 M (green trace); (**b**) K 2p region; (**c**) P 2p and Al 2s region.

X-ray photoelectron spectroscopy survey of all Pt SPE samples showed signals relevant to C 1s, O 1s, Pt 4f, Pt 4d Pb 4f, Pb 4s, and Al 2s, as depicted in Figure 2. Photopeaks related to K 2p and P 2p are present only on phosphate-LIFT treated samples (Figure 2, inset b and c).

In Table 1 the atomic percentage composition (At. %) of untreated Pt SPE and after LIFT with 0.1 M and 1 M phosphate buffer, as obtained from the XPS analysis is reported. The presence of C 1s dominated by the component at 285.0 eV is directly attributed to carbon contamination (C-H and C-C) coming from the XPS chamber and to the intrinsic sample composition as well as the two carbon oxidized components at 286.4 and 288.6 eV (Figure S1). However, after LIFT treatment a marked reduction of the intrinsic carbon content is observed (Table 1). Moreover, when K^+^ is present, the K 2p related signal (Figure S1 panel b) is detected and is characterized by two well resolved spin-orbit peak components: K 2p_3/2_ (293.0 eV) and K 2p_1/2_ (296.1 eV) [17]. In parallel, an evident increase of the oxygen content with a shift from 531.5 eV typical for metal oxides (i.e., Pt, Pb, and Al) to 532.0 eV (Figure S2), typical of phosphate oxygen, in LIFT treated samples is consistent with the presence of phosphate species transferred to the electrode surface. This oxygen species attribution is confirmed also by the parallel increase of oxygen and phosphorus atomic percentage correlated to the phosphate concentration used for the LIFT treatment. The analysis of untreated electrodes indicated the corresponding presence of Pb, Al and Pt at typical At. % of 4.3, 3.3, and 1.8, with an unexpectedly low Pt percentage. After the 0.1 M phosphate buffer treatment, the Pt, Pb, and Al content remain essentially constant, indicating that the marked surface enrichment in P and O species occurs specifically at expenses of removed intrinsic C. After the 1 M phosphate buffer treatment, the C content further decreases accompanied by an increase in O and P, and a decrease of Pt and Pb, while Al content remains constant.

**Figure 3:**
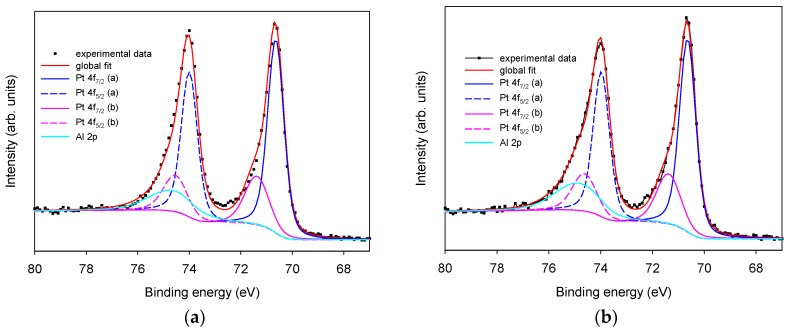
XPS high-resolution region of Pt 4f and Al 2p. Panel (**a**): untreated Pt SPE; panel (**b**): phosphate buffer 0.1 M LIFT treated Pt SPE.

X-ray photoelectron spectroscopy Pt 4f region is characterized by two well resolved spin-orbit peak components: Pt 4f_7/2_ and Pt 4f_5/2_, with a separation of 3.4 eV in Pt(0). In Figure 3, experimental data obtained using an untreated Pt SPE (panel a, dotted line) and after LIFT treatment with 0.1 M phosphate (panel b, dotted line) were reported for comparison. The HR X-ray photoelectron spectrum of all Pt 4f signals in these samples reveals the presence of the typical peak pair at 70.7 and 74.0 eV and spin-orbit separation of 3.30. eV, that can be attributed to Pt 4f_7/2_ and Pt 4f_5/2_ components [17]. However, the experimental data of analyzed samples can be fitted only using a pair of peaks with the most intense peak at binding energy 70.7 (Pt 4f_7/2_) and 74.0 (Pt 4f_5/2_) eV (Figure 3a,b, blue lines), and a less intense pair at 71.5 (Pt 4f_7/2_) and 74.5 (Pt 4f_5/2_) eV (Figure 3a,b, pink lines), both corresponding to Pt(0) present in two different phases of (i) pure and (ii) probably alloyed Pt [17,20]. The presence of Pt(II) oxidized components can be excluded, being their Pt 4f_7/2_ value of 72.40 eV [17]. No significant differences in the Pt oxidation state are observed for untreated Pt SPE and LIFT treated electrodes with 0.1 and 1.0 M phosphate (data not shown). The evident asymmetric shape of the high energy component of the Pt 4f_5/2_ signal cannot be explained in terms of the sole Pt contribution. In fact, the Pt 4f_5/2_ peak component must be theoretically the 75% of the Pt 4f_7/2_ intensity, because this fraction represents the degeneration of the 5/2 and 7/2 states. To optimize the curve fitting it is necessary to add a component at 74.6 eV attributed to Al 2p relevant to Al_2_O_3_ [17]. The presence of Al species is confirmed by the associated Al 2s signal at 119.0 eV as observed in Figure 2 (inset c)

**Figure 4:**
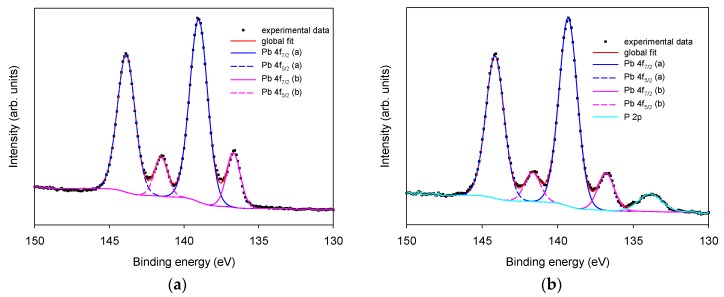
XPS high-resolution region of Pb and P 2p. Panel (**a**): untreated Pt SPE; panel (**b**): phosphate buffer 0.1 M LIFT treated Pt SPE.

Similarly, as shown in Figure 4, Pb 4f presents two pairs of peaks, with the most intense at 138.9 (Pb 4f_7/2_) and 143.8 (Pb 4f_5/2_) eV that can be attributed to Pb_3_O_4_ by comparing to the binding energy previously reported for Pb_3_O_4_ [21], and a less intense component at 136.6 (Pb 4f_7/2_) and 141.5 (Pb 4f_5/2_) eV attributed to Pb(0) [17]. In this region, the characteristic signal of P 2p can be observed only on phosphate buffer 0.1 M LIFT treated SPEs (panel (b)) at 133.8 eV typical of phosphate species [17]. Since SPEs are thoroughly rinsed with deionized water before XPS analysis, the persistence of the phosphate indicates its irreversible adsorption that induces a permanent chemical modification of the surface. The potassium present in the phosphate buffer 0.1 and 1 M LIFT treated samples is much less than the amount expected on the basis of phosphorus content, indicating that the phosphate is not transferred in the form of salt. A plausible hypothesis is that at least part of the phosphate combines with Pb to form Pb_3_(PO_4_)_2_, as indicated also by the increase of the oxidized form of Pb after LIFT treatment.

#### 3.1.2. Electrochemical Characterization

**Figure 5:**
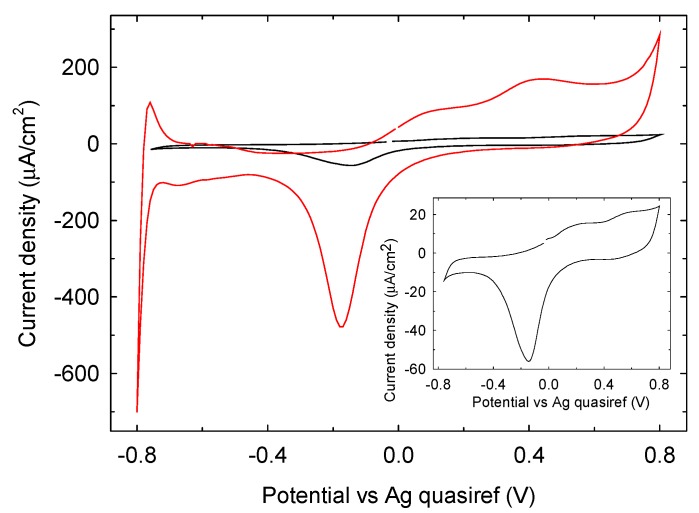
Cyclic voltammetry on untreated Pt SPE (black trace) and with phosphate buffer 0.1 M LIFT treated Pt SPE (red trace) in phosphate buffer 90 mM pH 7.0, scan rate 50 mV/s. Inset: zoom view of untreated Pt SPE.

The electrochemical characterization of Pt SPEs has been carried out by cyclic voltammetry (CV) in 90 mM phosphate buffer of pH 7.0 at a scan rate 50 mV/s, and the results obtained are shown in Figure 5. When a potential scan on Pt is recorded in the range −0.8 V and +0.8 V, typical Pt oxidation and reduction processes involving water/oxygen adsorbed species are observed in the region −0.4 V and +0.8 V [22,23]. The oxidation waves are shifted to more negative potential by 200 mV for the phosphate buffer 0.1 M LIFT treated sample (red trace) while the reduction wave is observed within a similar potential range obtained with an untreated Pt electrode (black trace). Noteworthy, after LIFT treatment with phosphate buffer 0.1 M the electrochemical response is markedly enhanced in terms of current intensity. A similar result is obtained with 1 M phosphate buffer (data not shown). Particularly important is the increase of the Pt activity in the hydrogen adsorption/desorption region between −800 and −400 mV. The charge collected in this region, Q_H_, was measured after double-layer correction, according to the literature [24]. A value of 6400 µC/cm^2^ was estimated and compared with the value of 210 µC/cm^2^ that represents the charge associated to the adsorption of a hydrogen monolayer on platinum [24]. This result indicates an enrichment in adsorbed hydrogen on phosphate buffer 0.1 M LIFT treated Pt, which is expected to enhance the glucose electrooxidation reactivity [25].

To investigate the possible reason for this selectively increased electroactivity, the electroactive area of the untreated and LIFT treated Pt electrodes, respectively, has been determined by studying the electroactivity of the [Fe(CN)_6_]^4−^/[Fe(CN)_6_]^3−^ redox couple. The electroactive area measurements, were estimated according to Randles–Sevcik equation (Equation (1)) by CV experiments at scan rate between 0.005 and 0.5 V s^−1^ in 10 mM [Fe(CN)_6_]^3−^ in 0.4 M KCl:

Ip = (2.69 × 10^5^)n^3/2^AD^1/2^C*v*^1/2^, (1)

where Ip is the peak current in amperes, n the number of exchanged electrons (*n* = 1 for ferricyanide/ferrocyanide red-ox pair), A the electrode area in cm^2^, D the diffusion coefficient (D = 7.60 × 10^−6^ cm^2^ s^−1^ for ferricyanide 10 mM in KCl 0.4 M [26]), C the concentration in mol cm^−3^ and *v* the sweep rate in V s^−1^ [27]. Surprisingly, the measured electroactive areas were found similar (8.1 × 10^−2^ cm^2^ for the untreated and 6.4 × 10^−2^ cm^2^ for the phosphate buffer 0.1 M LIFT treated electrodes) both obtained using 10 mM [Fe(CN)_6_]^3−^ in 0.4 M KCl. A possible explanation is that the transfer of phosphate on the electrode surface by LIFT enhances selectively the processes associated to oxygen and hydrogen.

**Figure 6:**
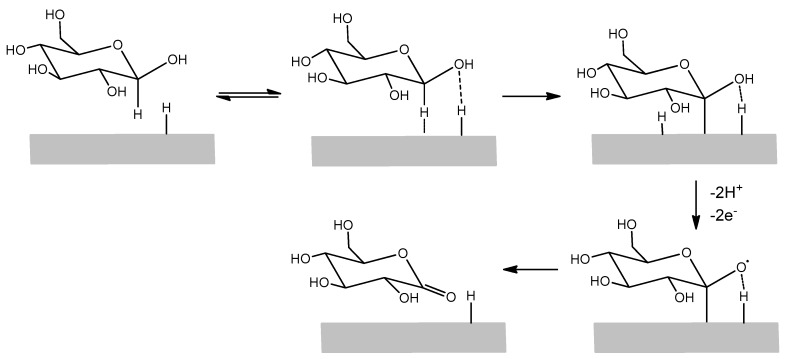
Simplified scheme of glucose electrooxidation mechanism on a Pt surface.

As anticipated, the enrichment of adsorbed hydrogen on phosphate buffer 0.1 M LIFT treated Pt (that favors the glucose electrooxidation) led us to test these modified SPEs for glucose detection. Indeed, in the hydrogen region there are single adsorbed hydrogen atoms on the surface that favor, in the accepted mechanism, the adsorption of β-glucose on the electrode surface. This mechanism is further depicted in Figure 6 [25]. The α-glucose anomeric form has a lower reactivity due to the unfavorable geometric orientation of the hydrogen bound to the C1 carbon. The subsequent step is the Pt extraction of the hydrogens attached to the C1 carbon and on the nearby -OH group together with their electrons, producing the glucono-δ-lactone, which is in turn hydrolyzed into gluconic acid. The result is an anodic current in a potential range where typically reduction processes take place. The oxidation peak current decreases as anions or organic species, particularly glucono-δ-lactone, are adsorbed on the electrode surface.

**Figure 7:**
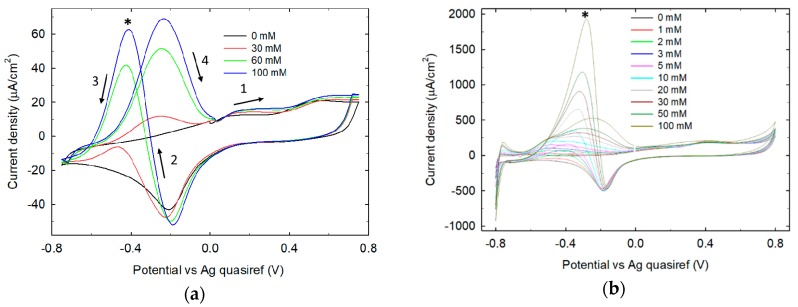
Glucose titration on untreated Pt SPE (**a**) and on phosphate buffer 0.1 M LIFT treated Pt SPE (**b**). The asterisks mark the upward peaks relevant to the cathodic sweeps, while arrows indicate the direction of potential sweeping.

The response of untreated Pt SPE and 0.1 M phosphate buffer LIFT treated Pt to increase glucose concentration is shown in Figure 7a,b respectively. As expected, untreated Pt cyclic voltammetry shows an evident response to glucose electrooxidation in the potential range −800/0 mV. Two current peaks of similar intensity (and increasing with glucose concentration) are observed, one belonging to the cathodic sweep (at around −420 mV) and the other to the anodic sweep (at around −240 mV). Peak positions and relative current intensities depend on the Pt surface properties (e.g., crystallinity), solution pH and possible anion adsorption [28]. On 0.1 M phosphate buffer LIFT treated Pt, two current peaks are observed in the same potential range, with significant differences in terms of potential peak position and relative current intensity. The cathodic sweep peak intensity shows a marked dependence on glucose concentration as well as its potential peak position shifting from −380 mV with 1 mM glucose to −296 mV with 100 mM glucose. The high ratio between the intensities of the cathodic and anodic sweep peaks indicates that the Pt surface is efficiently cleaned during the high potential scan (between 0 and +800 mV). This implies that the oxidation peak observed in the cathodic sweep starts on freshly reduced electrode surface and rises steeply when the hydrogen adsorption on platinum sets in. During the subsequent anodic sweep, the electrode surface is instead poisoned by the adsorbed glucose electrooxidation reaction intermediates (e.g., glucono-δ-lactone, CO_2_).

The response of LIFT treated Pt in terms of cathodic sweep peak current is ~30-fold increased with respect to untreated Pt SPE. The current density obtained with glucose 0.1 M is comparable to that usually obtained with pure Pt [25], pointing out an extreme reactivity of the treated electrode given its content in Pt of less than 2 At % as obtained by XPS analysis.

**Figure 8:**
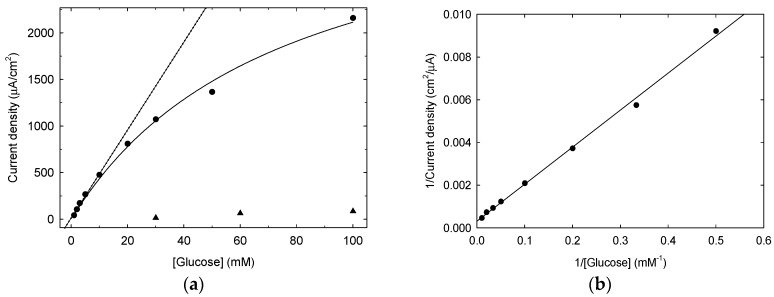
Panel (**a**): calibration curves for glucose detection on untreated Pt (triangles) and on phosphate buffer 0.1 M LIFT treated Pt (circles), the straight line interpolates the first 5 points (linear response) while the curved line (Langmuir model of Equation (2)) interpolates the data in the whole concentration range tested. Panel (**b**): double reciprocal plot of the calibration curve derived from data in panel (**a**).

Background corrected peak current at −0.4 V is used to establish a calibration plot for an untreated and a LIFT treated Pt SPE, as shown in Figure 8a. The poor response of the untreated Pt (triangles) is evident, while the LIFT-treated Pt (circles) shows a good linearity (r^2^ = 0.991, N = 5) in the glucose concentration range 1–10 mM. This makes this system useful for real-life applications as the expected average glucose level in human blood is in the 4–7 mM range. The response to glucose in the entire investigated range follows the same trend of a Langmuir adsorption model, in agreement with a role played by the surface coverage of the analyte in defining the current intensity. The experimental data can indeed be fitted (r^2^ = 0.995, *n* = 9) with the following equation [29]:

(2) $$i=kq_{e}=k\frac{Q_{0}bC_{Glu}}{1+bC_{Glu}}\;$$

in which *i* is the measured current, *q_e_* is the amount of glucose adsorbed on the Pt surface at equilibrium, *k* is a proportionality constant between *q_e_* and *i, Q*_0_ is the maximum monolayer coverage capacity of Pt, *b* is the Langmuir isotherm constant and C_Glu_ is the glucose concentration. The product *kQ*_0_ represents the maximum current *i_max_* at infinite glucose concentration.

Since the Langmuir model proves to well describe the sensor response in a wide concentration range, a rearrangement of Equation (2) can be performed aiming at obtaining a linear relationship, to be employed readily in the calibration step, when the glucose concentration to assess is expected to vary in a wide range:

(3) $$\frac{1}{i}=\frac{1}{kq_{e}}=\frac{1}{kQ_{0}}+\frac{1}{kQ_{0}bC_{Glu}}\;$$

Linearized data relevant to phosphate buffer 0.1 M LIFT treated Pt are presented in Figure 8b.

**Figure 9:**
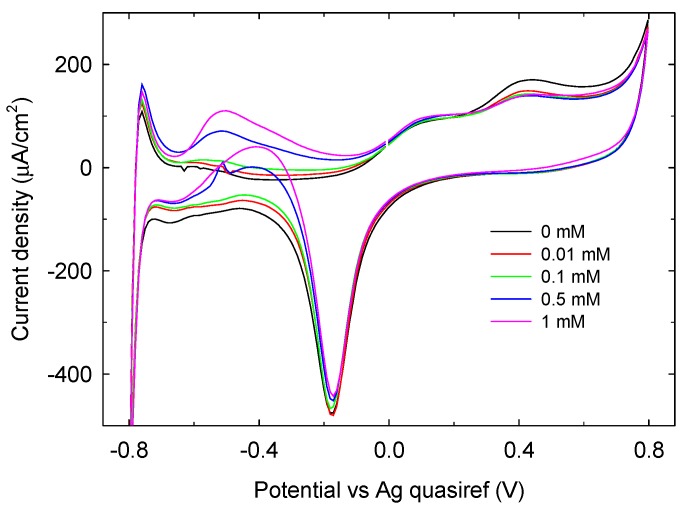
CV of phosphate buffer 0.1 M LIFT treated Pt SPE in the presence of glucose concentration ≤1 mM.

CV measurements have been conducted also at glucose concentration below 1 mM. As shown in Figure 9, a response distinguishable from the background (the difference is at least three times the signal to noise ratio) can be detected at glucose concentration as low as 10 µM, representing the measured limit of detection. On this basis, our sensing system can be considered competitive with other non-enzymatic glucose sensors using the electrochemical transduction [30,31,32].

### 3.2. LIFT Treatment of Au and GC SPEs

**Figure 10:**
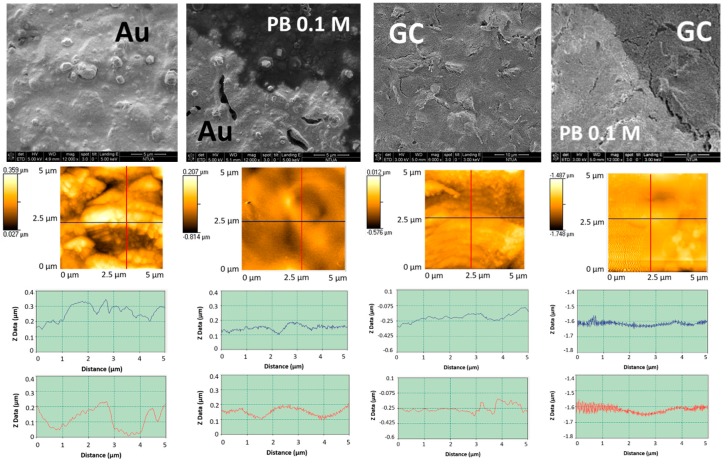
First row: SEM of Au and glassy carbon (GC) SPEs before and after LIFT treatment with phosphate buffer 0.1 M. Second row: corresponding atomic force microscopy (AFM) topographic images recorded in contact mode; third row: roughness profiles in the two orthogonal directions indicated in the AFM images.

The scanning electron micrographs and the atomic force micrographs obtained with Au and GC SPEs before and after LIFT treatment with 0.1 M phosphate buffer are shown in Figure 10. The surface roughness of the untreated Au and GC WEs resulted 55 nm and 95 nm, respectively as evidenced by AFM analysis. After the LIFT treatment the roughness of gold decreases to 36 nm while that of GC becomes 16 nm.

**Figure 11:**
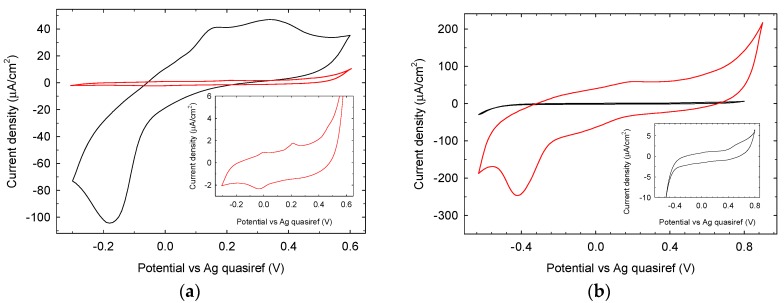
Cyclic voltammetry at (**a**) an untreated (black trace) and LIFT treated with 0.1 M phosphate buffer (red trace and inset) Au SPE and (**b**) an untreated (black trace and inset) and LIFT treated with 0.1 M phosphate buffer (red trace) GC SPE driven in phosphate buffer 90 mM pH 7.0, scan rate 50 mV/s.

The electrochemical characterization of Au and GC electrodes was based on the cyclic voltammograms obtained in 90 mM phosphate buffer (pH 7.0) at a scan rate of 50 mV/s. The voltammogram of untreated gold electrode (Figure 11a black trace) in the −0.3 V–+0.6 V range shows a characteristic reduction peak at −0.18 V and two oxidation peaks respectively at +0.15 V and +0.35 V. After LIFT treatment with 0.1 M phosphate buffer (red trace main graph and inset) the electrochemical properties are heavily influenced in terms of current reduction (95% decrease) and the reduction peak positively shifted to −0.03 V, while the oxidation peaks negatively shifted to +0.004 V and +0.22 V. These results show that the LIFT treatment with phosphate is detrimental for the electrochemical properties of gold SPE in the experimental condition used. The voltammogram of untreated GC electrode (Figure 11b black trace) in the range −650 mV/+900 mV, as expected, does not show any significant electrochemical activity. After LIFT treatment with 0.1 M phosphate buffer, the electrochemical response (red trace) is markedly enhanced in terms of current increase accompanied by the appearance of a reduction peak at −400 mV, possibly associated to the reduction of adsorbed oxygen formed in the anodic scan. Notwithstanding this enhanced reactivity, in the actual conditions, studies in the presence of glucose did not show any electroactivity as observed also for untreated GC SPE. These findings clearly demonstrate that the same LIFT treatment results in different modifications of the electrochemical response of SPEs depending strongly on the electrode material. Its chemical nature and the presence of specific contaminants (such as Pb in the case of Pt electrodes) play a crucial role in defining the surface chemical modifications triggered by the laser printing of a phosphate solution, which in turn confer the new specific electrochemical response.

## 4. Conclusions

It is well known that specific adsorptions of electroinactive species (i.e., Cl^−^, I^−^) on electrode surfaces can modify their electrochemical properties in enhancing or inhibiting oxidation and reduction processes. We have used the LIFT technique to permanently transfer, in physisorbed form, the phosphate anion on three typical electrode materials, namely Pt, Au, and GC. The induced effect of phosphate transfer by LIFT has been assessed. While from a morphological point of view all electrodes had a similar response, as they were flattened by the treatment, the electrochemical properties changed according to the different electrode material: the electroactivity of Au is inhibited while that of GC and Pt is greatly enhanced. The enrichment of hydrogen adsorbed layer on Pt electrode, promoted by the presence of bound phosphate, enabled its use as an effective glucose sensor, because the presence of hydrogen catalyzes glucose electrooxidation. The calibration plot in the 1–100 mM concentration range follows a Langmuir adsorption model, indicating that the rate limiting step of the electrochemical process is the glucose adsorption. These results encourage further work to permanently modify electrode surfaces by LIFT treatment with inorganic ions to investigate their emerging properties in sensing applications.

## Figures and Tables

**Table 1:** Atomic composition of untreated Pt SPE and after LIFT with 0.1 M and 1 M phosphate buffer.

	Pt SPE At. %	Pt SPE LIFT PB 0.1 At. %	Pt SPE LIFT PB 1 At. %
C 1s	59.5 ± 0.6	37.7 ± 3.0	30.1 ± 2.3
O 1s	31.0 ± 0.8	44.5 ± 2.6	52.4 ± 1.0
Pb 4f	4.3 ± 0.3	5.3 ± 0.2	3.5 ± 0.3
Pt 4f	1.8 ± 0.3	1.6 ± 0.7	0.5 ± 0.2
P 2p	0.0	6.8 ± 1.3	8.2 ± 0.8
Al 2s	3.3 ± 0.4	3.6 ± 0.5	3.7 ± 3.2
K 2p	0.0	0.5 ± 0.1	1.6 ± 0.3

## Supplementary Materials

The followings are available online at http://www.mdpi.com/2079-6374/8/4/91/s1, Figure S1: XPS high resolution region of C 1s and K 2p, Figure S2: XPS high resolution region of O 1s.
