# Supplementary material for: Phosphate Modified Screen Printed Electrodes by LIFT Treatment for Glucose Detection
Source: Biosensors (Basel). 2018 Oct 16;8(4):91. doi: 10.3390/bios8040091 (PMC6316885; doi:10.3390/bios8040091)
Supplement: Supplementary file 1 [file biosensors-08-00091-s001.pdf]

Supplementary Materials

Article

Phosphate Modified Screen Printed Electrodes by LIFT Treatment for Glucose Detection

Francesco Milano [1], Livia Giotta [2], Daniela Chirizzi [3], Simos Papazoglou [4], Christina Kryou [4], Annarita De Bartolomeo [5], Vincenzo De Leo [1,6], Maria Rachele Guascito [2,3,*] and Ioanna Zergioti [4]

[1] Istituto per i Processi Chimico Fisici, UOS Bari, Via Orabona 4, 70126 Bari, Italy; francesco.milano@cnr.it (F.M.); v.deleo@ba.ipcf.cnr.it (V.D.L.)
[2] Dipartimento di Scienze e Tecnologie Biologiche e Ambientali, Università del Salento, S.P. Lecce-Monteroni, 73100 Lecce, Italy; livia.giotta@unisalento.it
[3] IZS Puglia e Basilicata, U.O. Putignano. Via Chiancolla 1, C.da. S. Pietro Piturno, 70017 Putignano (BA), Italy; daniela.chirizzi@izspb.it
[4] Department of Physics, National Technical University of Athens, Iroon Polytehneiou 9, Zografou, 15780 Athens, Greece; simpap@mail.ntua.gr (S.P.); chkryou@central.ntua.gr (C.K.); zergioti@central.ntua.gr (I.Z.)
[5] Dipartimento di Beni Culturali, Università del Salento, S.P. Lecce-Monteroni, 73100 Lecce, Italy; annarita.debartolomeo@unisalento.it
[6] Dipartimento di Chimica, Università di Bari "Aldo Moro", Via Orabona 4, 70125 Bari, Italy
* Correspondence: maria.rachele.guascito@unisalento.it; Tel.: +39-0832-297075

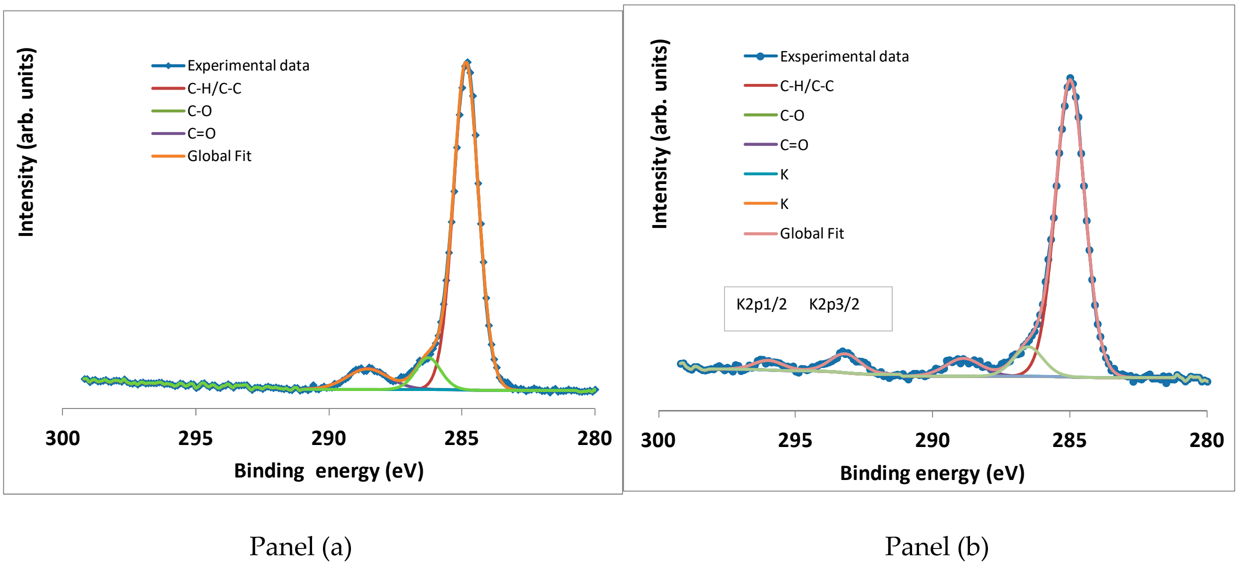

Figure S1. XPS high resolution region of C 1s and K 2p. Panel (**a**): untreated Pt SPE; panel (**b**): phosphate buffer 0.1 M LIFT treated Pt SPE.

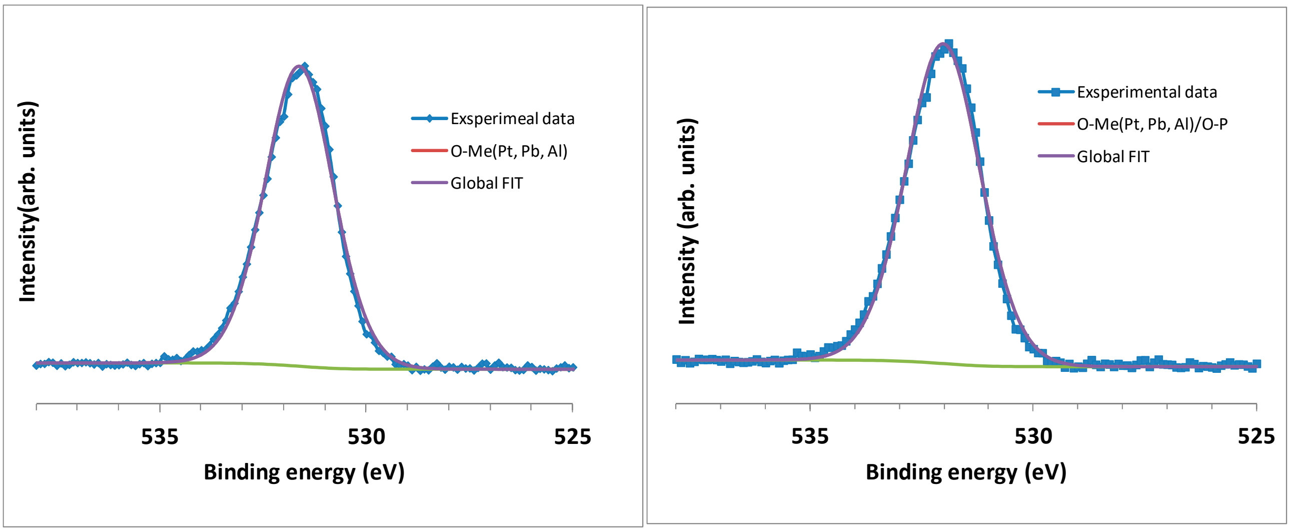

Panel (a) Panel (b)

Figure S2. XPS high resolution region of O 1s. Panel (**a**): untreated Pt SPE; panel (**b**): phosphate buffer 0.1 M LIFT treated Pt SPE.
